# Biological Impact of True-to-Life PET and Titanium-Doped PET Nanoplastics on Human-Derived Monocyte (THP-1) Cells

**DOI:** 10.3390/nano15131040

**Published:** 2025-07-04

**Authors:** Aliro Villacorta, Michelle Morataya-Reyes, Lourdes Vela, Jéssica Arribas Arranz, Joan Martín-Perez, Irene Barguilla, Ricard Marcos, Alba Hernández

**Affiliations:** 1 Group of Mutagenesis, Department of Genetics and Microbiology, Faculty of Biosciences, Universitat Autònoma de Barcelona, 08193 Cerdanyola del Vallès, Spain; herlem.morataya@uab.cat (M.M.-R.); lulis002@hotmail.com (L.V.); jessica.arribas@uab.cat (J.A.A.); juan.martin.perez@uab.cat (J.M.-P.); irene.barguilla@uab.cat (I.B.); ricard.marcos@uab.cat (R.M.); 2 Facultad de Recursos Naturales Renovables, Universidad Arturo Prat, Iquique 101000, Chile; 3 Faculty of Health Sciences Eugenio Espejo, Universidad UTE, Quito 170508, Ecuador

**Keywords:** nanoplastics, polyethylene terephthalate (PET), tru-to life MNPLs

## Abstract

In the environment, plastic waste degrades into small particles known as microplastics and nanoplastics (MNPLs), depending on their size. Given the potential harmful effects associated with MNPL exposure, it is crucial to develop environmentally representative particles for hazard assessment. These so-called true-to-life MNPLs are generated through in-house degradation of real-world plastic products. In this study, we produced titanium-doped nanoplastics (NPLs) from opaque polyethylene terephthalate (PET) milk bottles, which contain titanium dioxide as a filler. The resulting PET(Ti)-NPLs were thoroughly characterized using scanning electron microscopy (SEM), energy-dispersive X-ray spectroscopy (EDS), mass spectrometry (MS), dynamic light scattering (DLS), ζ-potential measurements, transmission electron microscopy (TEM), and Fourier-transform infrared (FTIR) spectroscopy. Human-derived THP-1 monocytes were employed to investigate particle uptake kinetics, dosimetry, and genotoxicity. A combination of flow cytometry and inductively coupled plasma mass spectrometry (ICP-MS) enabled the quantification of internalized particles, while the comet assay assessed DNA damage. The results revealed dose- and time-dependent effects of PET(Ti)-NPLs on THP-1 cells, particularly in terms of internalization. Titanium doping facilitated detection and influenced genotoxic outcomes. This study demonstrates the relevance of using environmentally representative nanoplastic models for evaluating human health risks and underscores the importance of further mechanistic research.

## 1. Introduction

The growing accumulation of plastic waste in the environment has become a major global concern. Of relevance is the widespread presence of plastic degradation products, broadly termed microplastics (MPLs), which pose potential health risks that remain poorly understood [1]. First introduced by Thompson et al. [2], the term “microplastic” typically refers to plastic fragments smaller than 5 mm. While this definition remains operational, it has been widely debated [3], as it inadequately accounts for the critical role of particle size in biological interactions. Smaller particles have a higher likelihood of being internalized by living organisms, including humans. To address this, we adopt a size-based classification in which nanoplastics (NPLs) range from 1 to 1000 nm and MPLs from 1 to 1000 µm [4]. Environmental degradation of plastic waste, driven by various abiotic and biotic factors, produces a heterogeneous mixture of micro- and nanoplastic particles (MNPLs). As degradation progresses, the mixture becomes increasingly diverse in size, necessitating the use of the inclusive term micro/nanoplastics (MNPLs) to reflect the continuum of particle sizes found in environmental samples.

Beyond particle size, other features—such as polymer type, additives, and shape—also influence MNPL toxicity. While numerous studies have recently explored the potential adverse effects of MNPLs [5,6], most have used commercially available, pristine polystyrene MNPLs (PS-MNPLs) due to their accessibility. However, for more realistic risk assessments, there is a critical need to evaluate environmentally representative MNPLs generated from actual plastic waste. To address this, true-to-life MNPLs have been proposed [7]. These particles are produced by in-house degradation of consumer plastic items using mechanical methods, resulting in (1) polymeric compositions reflective of real environmental pollutants, (2) diverse morphologies and sizes, (3) the inclusion of additives used in commercial products, and (4) sufficient yields for comprehensive biological testing in vitro and in vivo.

Several studies have applied mechanical sanding using a rotary Dremel tool to produce true-to-life MNPLs from plastic items such as PET water bottles, HDPE containers, PP glasses, and PS utensils [4,8]. Recently, this approach was extended to opaque PET milk bottles, which are pigmented with titanium dioxide (TiO_2_) nanoparticles (TiO_2_-NPs) to reduce gas permeability and protect UHT milk from light [9]. This process yields titanium-doped PET nanoplastics [PET(Ti)-NPLs], where TiO_2_-NPs are embedded within the PET matrix and remain stable over time. These particles hold potential for tracking plastic fate in biological systems, as shown in recent *Drosophila* studies [10].

Initial characterization of PET(Ti)-NPLs revealed no overt toxicity in three human hematopoietic cell lines (THP-1 monocytes, TK6 lymphoblasts, and Raji-B lymphocytes), though THP-1 cells displayed the highest internalization rate [9]. To expand on these findings, we further investigated PET(Ti)-NPLs in THP-1 monocytes, focusing on internalization kinetics and genotoxicity. Titanium doping enables precise quantification of particle uptake via inductively coupled plasma mass spectrometry (ICP-MS). Genotoxicity was assessed using the comet assay to detect DNA strand breaks. For comparison, NPLs derived from PET water bottles [4] and commercial TiO_2_-NPs were also tested.

## 2. Material and Methods

### 2.1. Particle Source and Characteristics

To obtain environmentally representative PET(Ti)- and PET-NPLs from commercially available sources, diary opaque and regular water PET bottles were used as the source of nanoplastics by following previously published procedures [4,9]. Briefly, in two different procedures, the opaque water and milk bottles were fractioned into smaller pieces of 12 cm^2^. These smaller fractions were then sanded with a Dremel 3000 electric multitool with a flexible extension attached to a diamond rotary burr. All components were from Dremel (Robert Bosch Tool Corporation, Mount Prospect, IL, USA). Film-sanded powder was sieved though a 0.20 mm mesh CISA R-92 (Allegion, Dublin, Ireland) until 4 g were obtained. The sieved material was then dispersed for 2 h on a 150 mL beaker containing 40 mL of 60 °C pre-heated 90% *v*/*v* trifluoroacetic acid (TFA) (Sigma-Aldrich, St. Louis, MO, USA) for 2 h under constant stirring on a stir heating plate (Heidolph Instruments GmbH & Co. KG, Schwabach, Germany). After 2 h, the temperature was lowered to room temperature and the sample was kept on agitation overnight. On the second day, 40 mL of 20% *v*/*v* TFA was added, big aggregates were physically disaggregated or eliminated, and the generated suspension was then kept on constant stirring for 24 h. Particle suspensions were then passed through the 0.2 mm sieve to eliminate any bigger pieces, and then the collected material was distributed in 10 mL glass tubes and centrifuged at 2500 rcf for 1 h on an Eppendorf 5810 R (Eppendorf AG, Hamburg, Germany). Once the particles were deposited, the TFA acid was eliminated and the particles resuspended in a final volume of 400 mL of 0.5% sodium dodecyl sulfate (SDS) (Merck KGaA, Darmstadt, Germany). The total volume was homogenized by pipetting and distributed in two 250 mL beakers prior to being ultrasonicated in a cold-water bath for 2 min with 9/9 s cycles of 25% amplitude sonication/break on an SSE-1 Branson sonicator (Branson Ultrasonics Co., Brookfield, CT, USA). The sonicated particle suspension was then transferred to a 200 mL graduated cylinder, and bigger particles were then left to sediment for 1 h. The top 100 mL of each cylinder was then collected and distributed in 50 mL tubes and centrifuged under the same conditions as before to eliminate SDS. Particle pellets were then resuspended and transferred to the previously weighed 10 mL glass tubes, and then washed twice with water and twice with ethanol and left to dry for two days under an air laminar flow hood. The particles were then resuspended in Milli-Q water to a final stock concentration of 10 mg/mL and sonicated for 16 min at 10% amplitude in a cold-water bath. One mL of aliquots were then prepared, immediately frozen in liquid nitrogen, and stored at −80 °C. TiO_2_ particles were purchased from Chemours^TM^ (Washington, DE, USA). For all biological applications, particles were suspended using the Nanogenotox protocol [11].

Nanoparticle tracking analysis. Size distribution for all particles in suspension was complementarily determined by a combination of both light scattering and Brownian movement using a Nanosight NS300 from Malvern Panalytical Ltd. (Cambridge, United Kingdom). Briefly, a stock solution of 10,000 µg/mL of each nanoplastic/particle suspensions (PET, PET(Ti), and TiO_2_) was sonicated for 30 min in an ultrasonic bath (Vevor, Houston, TX, USA). A suspension of 100 µg/mL was prepared in Milli-Q water, and right before the measurement, a 1:50 dilution was carried out using Milli-Q water in an air-pressured cleaned 20 mL glass vial and then vigorously mixed in a SA8 vortex (Merck KGaA, Darmstadt, Germany) operated at 1000 RPM. One mL of the diluted suspension was then transferred to the Nanosight microfluidic system with a syringe. The injection was carried out at decreasing speeds from 1000 to 50 a.u., and three independent measurements were conducted following this procedure. Data were acquired/recorded using a sCMOS detector (Oxford Instruments, Oxford, UK) with a 488 nm laser setup.

Scanning electron microscopy and electron dispersion spectroscopy (SEM-EDS).

PET(Ti)-NPL suspensions were prepared at concentrations of 200 µg/mL from the previously prepared 10,000 µg/mL nanoplastic dispersions. From the diluted aliquot, 10 µL was pipetted on 5 × 5 mm precut silicon chips (Ted Pella, Inc., Altadena, CA, USA) and dried overnight on a Petri dish internally covered with filter paper inside a laminar air flow hood. Particles were then analyzed with a scanning electron microscope (Sem Zeiss, Oberkochen, Germany). Fields were aleatorily selected, and the EDS spectra were obtained with a X-Max 20 mm EDS system (Oxford Instruments, Oxford, UK) and analyzed by INCA Energy software (INCA, Grinnell, IA, USA). Complementary dynamic light scattering (DLS), ζ-Potential, transmission electron microscopy (TEM), and Fourier transform infrared spectroscopy (FTIR) studies are presented in the Supplementary Materials (Section S1 and Figure S1).

### 2.2. Cell Culture

The human leukemia monocytic THP-1 cell line was used. THP-1 cells were grown in filtered cap T-25 flasks (SPL Life Sciences Co., Pocheon-si, Gyeonggi-do, Republic of Korea) at cell concentrations ranging from 0.5 × 10^6^ to 1 × 10^6^ and at a maximum volume of 5 mL. Cells were grown in suspension in RPMI (Roswell Park Memorial Institute) culture medium supplemented with 10% FBS (fetal bovine serum), 1% glutamine (Biowest, Nuaille, France), and 2.5 µg/mL of Plasmocin^®^ (InvivoGen, San Diego, CA, USA). Cultures were grown at 37 °C in a humidified atmosphere with 5% CO_2_ in an ICO150med CO_2_ incubator (Memmert GmbH + Co. KG, Schwabach, Germany).

### 2.3. Cell Viability

To determine the effects of PET(Ti)-NPLs in THP-1 cells, 5 × 10^5^ cells/mL were seeded in U-bottom 96-well plates in a final volume of 0.2 mL of medium and treated with NPLs at concentrations of 0 to 100 µg/mL for 24 h. Exposure to the same concentrations of PET-NPLs and the corresponding TiO_2_-NP concentrations was carried out in parallel, as was seeding of non-treated THP-1 control cells. After the exposure time, the cells were mixed and diluted at 1:100 in ISOFLOW, and the cell number was determined using a ZTM Coulter Counter (Beckman Coulter Inc., Brea, CA, USA). The average cell number for each NPL concentration was compared with the average number of non-treated THP-1 cells after 24 h. Data were analyzed using GraphPad Prism 8.0.2. (GraphPad Software Inc., Boston, MA, USA).

### 2.4. Internalization Studies

#### 2.4.1. Flow Cytometry Fluorescent Particle Detection

THP-1 cells were seeded at an initial concentration of 5 × 10^5^ cells/mL in U-bottom 96-well plates (200 µL/well) and exposed for 24 h to iDye-labeled PET(Ti)-NPLs. The protocol to dye MNPLs using the textile dye iDye PolyPink (Rupert, Gibbon & Spider, Inc., Healdsburg, CA, USA) was recently published [12]. The tested concentrations were 0, 25, 50, and 100 µg/mL. For the flow cytometry analysis, exposed cells were separated by centrifugation (8 min at 300 rcf) using Eppendorf 5810 R tubes (Eppendorf AG, Hamburg, Germany) and rinsed twice with sterilized phosphate-buffered saline (PBS 1×) (Gibco, Thermo Fisher Scientific Inc., Waltham, MA, USA) prepared in distilled water. Cell data were collected on a CytoFLEX LX device (Beckman Coulter, Brea, CA, USA) and analyzed using the CytExpert 2.5 software from the same company. Data were analyzed using GraphPad Prism 8.0.2. (GraphPad Software Inc., Boston, MA, USA), and charts were prepared by using the aforementioned software and the Excel^®^ program (Microsoft, Redmond, WA, USA).

#### 2.4.2. Internalization Kinetics and Cell Complexity Analysis

THP-1 cells, at a cell density of 5 × 10^5^ cells/mL, were grown on 96-well plates at a final volume of 200 µL/well and exposed to a concentration of 100 µg/mL of PET(Ti)-NPLs for a period ranging from 1 to 24 h. Exposed cells were evaluated in static conditions and on a digital Rocker 3D (IKA-Werke GmbH & Co, Staufen, Germany). In parallel, cells were exposed to 3 and 100 µg/mL of TiO_2_ nanoparticles. For all timepoints, data were acquired in three independent experiments in triplicate for treated and control groups. Internal cell complexity, as an indicator of internalization, was determined as the percentage of positive cells (more complex) and the increase in internal complexity (how complex) by side scatter analysis using a CytoFLEX LX device (Beckman Coulter, Brea, CA, USA) and analyzed on CytExpert 2.5 from the same company. Data were analyzed and charts were prepared using GraphPad Prism 8.0.2. (GraphPad Software Inc., Boston, MA, USA).

#### 2.4.3. Confocal Microscopic Imaging

THP-1 cells were grown on U-bottom 96-well plates at a total volume of 200 µL/well for 24 h. The experiment used a concentration of 100 µg/mL for iDye PET-NPLs and IDye PET(Ti)-NPLs, while the concentration of 3 µg/mL was used for TiO_2_NPs. As for fluorescence detection by flow cytometry, once exposed, the cells were washed twice using 1x PBS and resuspended in pre-warmed supplemented culture media. A total of 300 µL of the respective suspensions were transferred to each well of a chambered coverslip with 8 wells (ibid GmbH, Gräfelfing, Germany) for confocal microscopy observation. Cell nuclei were labeled using Hoechst 33342 (Sigma-Aldrich, Madrid, Spain) with an excitation wavelength (eWL) of 405 nm and an emission collected wavelength (ecWL) of 415–503. Cell membranes were labeled using Cellmask (eWL 633 nm and ecWL of 645–786). For iDye-labeled PET- and PET(Ti)-NPLs, an eWL of 561 nm and an ecWL of 570–630 were used. For TiO_2_ localization, reflection images were obtained in parallel in all samples. The confocal device utilized was a Leica TCS SP5 confocal microscope, and the image process was carried out using ImageJ 1.54f analysis software, version 1.8.0_322 (USA National Institutes of Health, Bethesda, MD, USA).

#### 2.4.4. Scanning and Transmission Electron Microscopy

THP-1 cells at a cell density of 5 × 10^5^ cells/mL were grown in filtered cap T-25 flasks (SPL Life Sciences Co., Pocheon-si, Gyeonggi-do, Republic of Korea) in standard conditions. Exposure to PET(Ti)-NPLs lasted for 24 h at a concentration of 100 µg/mL. This concentration was selected according to previous studies [9]). Exposed and control cells were collected and fixed at 4 °C in aldehyde mix (2% paraformaldehyde and 2.5% glutaraldehyde) and subjected to a post treatment with 1% osmium tetroxide (OsO_4_) and 0.8% potassium hexacyanoferrate (II) trihydrate. The following steps included dehydration in successive steps of increasing concentrations of acetone (as detailed in Supplementary Table S1) as a previous step for resin infiltration at room temperature (as detailed in Table S2). The resin sample mixture was then deposited in molds for sample block creation. Thin and ultrathin (70 nm) sections were prepared on a Leica EM UC6 ultramicrotome, and the sections were placed on AMR carbon support film on a copper 200-square mesh with and without carbon covering for scanning electron microscopy (SEM, Zeiss MERLIN FE-SEM, Oberkochen, Germany) and transmission electron microscopy (TEM, JEOL JEM 1400 instrument JEOL LTD, Tokyo, Japan), respectively. SEM visualization was complemented by elemental energy dispersive spectroscopy (EDS) detection using an X-Max 20 mm EDS system (Oxford Instruments, Oxford, UK). TEM was complemented with electron diffraction detection using a CCD GATAN Orius detector (Gatan, Inc., Pleasanton, CA, USA).

### 2.5. Cell Sorting and Titanium Quantification

Similar to the cell culture maintenance strategy, a final volume of 4 mL of suspension of THP-1 cells at a cell density of 5 × 10^5^ cells/mL were grown in standard conditions for 24 h under an exposition regime of PET(Ti)-NPLs at a concentration of 100 µg/mL in filtered cap T-25 flasks (SPL Life Sciences Co., Pocheon-si, Gyeonggi-do, Republic of Korea). Cells were then collected and transferred to 15 mL tubes. Untreated (control) cells were used to diagram the gates for cell sorting of the treated cells. Exposed cell populations were sorted using a BD FACSJazz™ Cell Sorter and analyzed using the BD FACS Software version 6.0 sorter from the same company (Becton, Dickinson & Co., Franklin Lakes, NJ, USA). Positive and negative cells were collected in 15 mL tubes, and the relative Ti content was determined by ICP-MS. Shortly thereafter, the cell suspensions were kept at −20 °C, and before the measurement they were thawed and vigorously homogenized for approximately 1 min by vortexing. The collected aliquots were subjected to chemical digestion using HNO_3_ 65% p/v and HF 40% in a 4:1 ratio. Samples were then diluted in HNO_3_ 1% *v*/*v* and analyzed on an ICP-MS Agilent, model 7900 device (Agilent Technologies, Santa Clara, CA, USA).

### 2.6. Effects on ROS Levels

Reactive oxygen species (ROS) generation in THP-1 cells following nanoplastic (NPL) internalization was assessed using the dihydroethidium (DHE; Calbiochem, San Diego, CA, USA) assay. THP-1 cells were seeded at a density of 5 × 10^5^ cells per well in U-bottom 96-well plates (triplicate) with a final volume of 0.2 mL RPMI medium and subsequently exposed to increasing concentrations (0–100 µg/mL) of PET(Ti)-NPLs and TiO_2_-NPs for 24 h. Following exposure, cells were centrifuged at 1200 rpm for 8 min, washed with PBS 1X, and stained with DHE at a final concentration of 1 µg/mL. Samples were incubated at 37 °C for 30 min prior to analysis. ROS production was quantified by flow cytometry (CytoFLEX LX, Beckman Coulter, Brea, CA, USA) using CytExpert software, measuring mean FIT-C fluorescence in 10,000 single-cell events per condition. Average mean fluorescence in the treated cells was compared against that of non-treated THP-1 cells, and data were analyzed using GraphPad Prism 8.0.2 (GraphPad Software, San Diego, CA, USA).

### 2.7. Genotoxicity

The comet assay. The levels of DNA damage in THP-1 cells exposed for 24 h to PET-NPLs, PET(Ti)-NPLs, and TiO_2_NPs were evaluated by using the single-cell gel electrophoresis (comet) assay, which detects mainly single-strand breaks. The cells (exposed/unexposed) were centrifuged at 0.30 RCF for 8 min at 4 °C. Pelletized cells were washed with cold PBS 1x, and the concentration was adjusted to 1 × 10^6^ cell per mL. The cells were then mixed 1:10 with 0.75% previously melted low-melting-point agarose at a stabilized temperature of 37 °C, carefully mixed, and then dropped in triplicate onto GelBond^®^ films (GBFs) (Life Sciences, Vilnius, Lithuania). GBFs with the samples were kept overnight in cold lysis buffer at 4 °C and, finally, washed. An incubation step lasting for 30 min at 4 °C on the electrophoresis buffer was carried out before submission to electrophoresis (20 V, 300 mA, 4 °C, 23 min). GBFs were then recovered and washed twice with cold PBS 1x, fixed in absolute ethanol for at least 1 h, and air-dried overnight at room temperature. A posterior staining with SYBR Gold (Invitrogen, Thermo Fisher Scientific Inc., Waltham, MA, USA) was carried out for 20 min on a Heidolph orbital shaker (Heidolph Instruments GmbH & Co., Schwabach, Germany). GBFs were then mounted and covered on glass and analyzed by epifluorescent microscopy (Olympus BX50, Hamburg, Germany). The levels of DNA damage, determined as percentage of DNA in the tail, were quantified using the Komet 5.5 Image analysis system (Kinetic Imaging Ltd., Liverpool, UK). Data were analyzed and graphics were prepared using GraphPad Prism 8.0.2. (GraphPad Software Inc., San Diego, MA, USA).

## 3. Results and Discussion

### 3.1. Nanoparticle Characteristics

**Figure 1:**
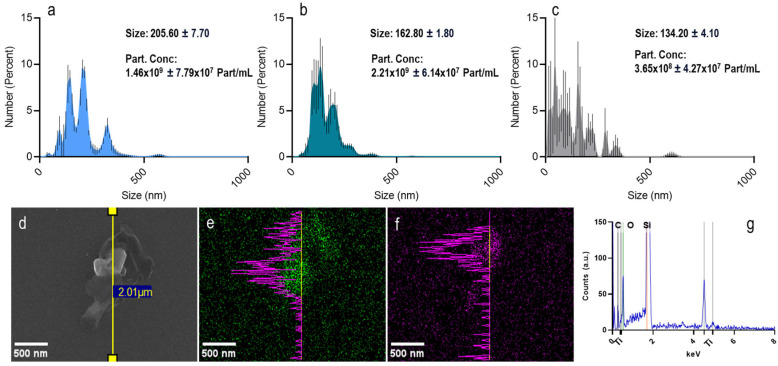
Nano-tracking analysis of PET-NPLs (**a**), PET(Ti)-NPLs (**b**), and TiO_2_NPs (**c**) expressed as a percentage with standard error. SEM images of the PET(Ti) composite (**d**) and the mapping for Ti (**e**) and carbon (**f**) along with the EDS spectra (**g**), confirming the presence of Ti inside what at first glimpse seems to be only PET particles.

The PET- and PET(Ti)-NPLs employed in the present study were exhaustively characterized. One of the implemented methodologies was particle-by-particle tracking, and, as observed in Figure 1, both the sizes and the concentrations were consistent and fell within comparable ranges and concentrations. It should be noted that, in addition to the NPLs, TiO_2_NPs were also included in the study to cover as many variables as possible (Figure 1a–c). Moreover, it was observed that TiO_2_NPs could be detected within the PET(Ti)-NPLs, and it was feasible to perform compositional mapping. This not only facilitated biological tracking but also enabled particle-level monitoring, thereby confirming the mixed nature of the material under study (Figure 1d–f). Furthermore, analysis of the mapping regions for titanium confirmed its presence via energy-dispersive spectroscopy (EDS), which revealed not only the characteristic peaks for carbon and oxygen from the polymer but also those indicative of titanium (Figure 1g). Additionally, the hydrodynamic behavior of the particles was examined, revealing a coherent size distribution of 282 ± 3.12 nm for PET- and 320 ± 8.18 nm for PET(Ti)-NPLs, with polydispersity indexes of 0.47 and 0.34, respectively. The correlation curves are also presented (Figure S1b,c,e,f). Transmission electron microscopy, presented in the same figure (Figure S1a,d), further demonstrated the similar nature of the generated nanoplastics and underscores the importance of complementary analytical techniques in characterizing not only the surface but also the internal composition of the particles. Finally, to determine the chemical nature of the polymer, Fourier-transform infrared spectroscopy (FTIR) data show the previously described characteristic peaks for PET [13,14,15,16], provided in the Supplementary Materials (Figure S1g).

The above data confirm the usefulness of the presented protocol to obtain true-to-life PET MNPLs, as evidenced by recent publications [10,12,17,18,19,20,21].

### 3.2. NPL Cytotoxicity

**Figure 2:**
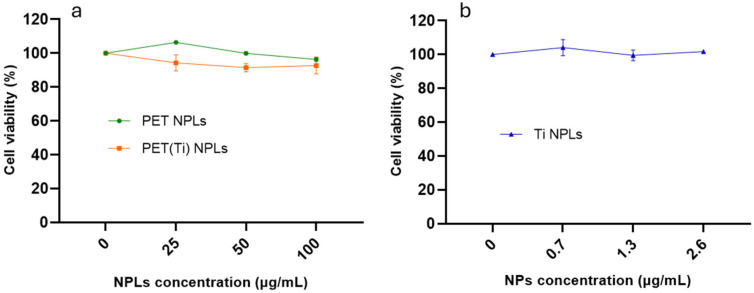
Cell viability after 24 h of THP-1 exposure to 0–100 µg/mL of PET- and PET(Ti)-NPLs (**a**) and the corresponding concentration of TiO_2_-NPs in the PET(Ti)-NPLs (0–2.6 µg/mL) (**b**). Values of cell viability were normalized against non-treated THP-1 cell number after 24 h.

For the selection of the concentrations to be used, a previous cytotoxicity study was required. Our results revealed no distinct cytotoxic patterns between PET- and PET(Ti)-NPLs in THP-1 monocytes, at any of the concentrations studied. The highest concentration (100 µg/mL) of both PET- and PET(Ti)-NPLs caused significant biological observable effects in terms of cell viability (Figure 2a). At the same time, concentrations of TiO_2_-NPs at maximum present concentrations in PET(Ti)-NPLs and seriated dilutions revealed no statistically significant cytotoxicity (Figure 2b). The results for these nanoplastics are in line with previous reports for acute exposure in several cell lines studied for both materials [9,18,21,22].

### 3.3. Internalization of PET(Ti)-NPLs

The kinetics of cell uptake of MNPLs is a complex topic since it depends not only on the physicochemical characteristics of the MNPLs but also on the cell used. Using different hematopoietic cell types, a differential cell uptake was observed for the different cells [5,23]. In the same way, using PS-NPLs with different surface functionalization, different dynamics of internalization have been observed [24]. This highlights the relevance of accurately confirming the cell uptake of the used MNPLs, mainly when new true-to-life MNPLs are evaluated.

**Figure 3:**
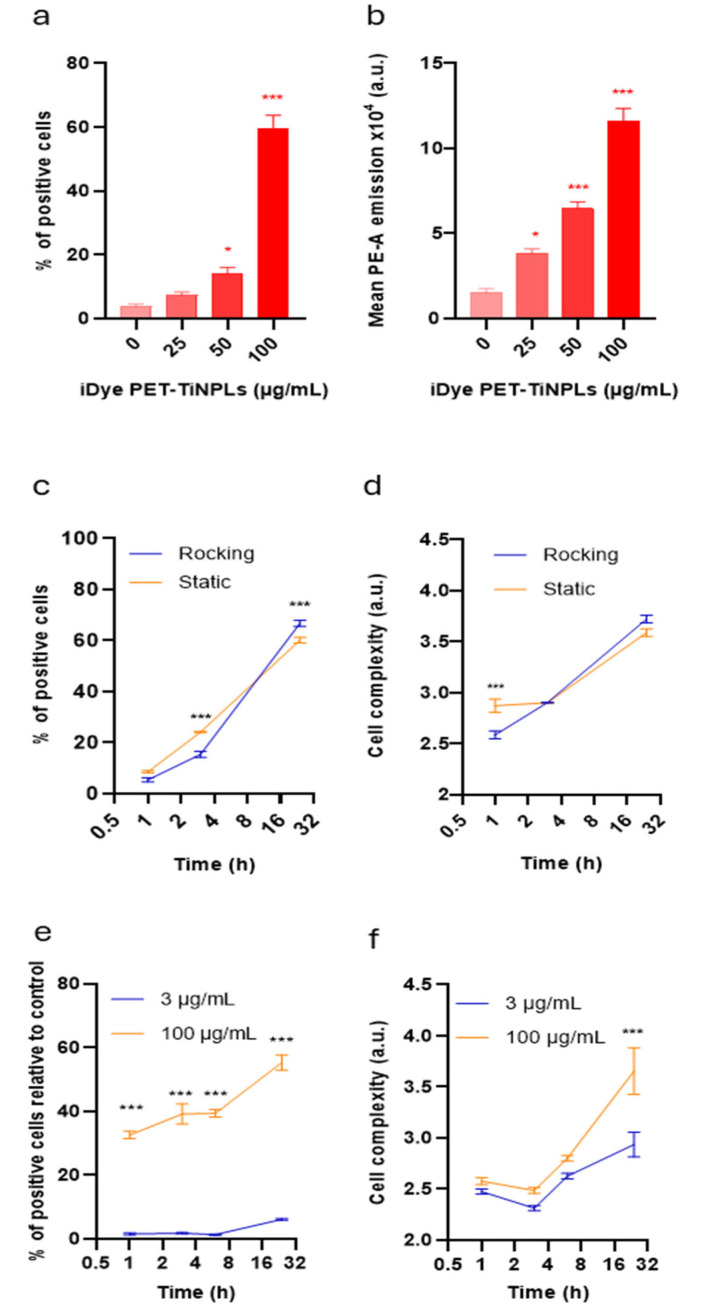
Relative internalization of PET(Ti)-NPLs in THP-1 cells exposed to 25 to 100 µg/mL lasting for 24 h. The percentage of cells that internalized PET(Ti)-NPLs (**a**) and the relative quantity of particles internalized (**b**). The effects of internalization (100 µg/mL) using rocking and static platforms are indicated for the percentage of cells with PET(Ti)-NPLs (**c**) and the quantity of internalized particles (**d**). Differences in internal complexity in terms of percentage of cells that internalized PET(Ti)-NPLs (**e**) and the relative quantity of particles internalized (**f**) of two different concentrations of TiO_2_NPs in a time range of exposure of 1 to 24 h. * *p* ≤ 0.05; *** *p* ≤ 0.001.

In this study, we analyzed the dynamic of internalization (cell complexity) by light scattering, as well as the levels of fluorescence intensity within the cells. We chose THP-1 as a suitable cell mode due to its demonstrated MNPL uptake [25] and exposure conditions of 25, 50, and 100 µg/mL lasting for 24 h. The results obtained are indicated in Figure 3. Under these conditions, approximately 60% of the cell population internalized PET(Ti)-NPLs (Figure 3a), which agrees with previous studies [9]. In addition to the percentage of cells with internalized PET(Ti)-NPLs, the amount of internalization was also determined (Figure 3b). It must be noted that within the population of cells that internalized PET(Ti)-NPLs there were subpopulations with higher emission intensities, allowing us to distinguish between cells with more or fewer particles. As observed, the proportion of cells with high levels of internalization was concentration dependent. This approach of measuring exposure is interesting because it has been shown that the levels of internalization correlate with the induced effects [25]. To determine whether exposure conditions affected internalization, the effects of a rocking platform that kept the cells in constant motion were compared with those of static exposure. The results show that both conditions induced similar proportions of cell internalization and similar levels of internalization (Figure 3c,d). Interestingly, this trend remained regardless of the length of exposure to PET(Ti)-NPLs in the cell type evaluated, although the internalization was time dependent.

An attractive question is whether the presence of titanium in PET(Ti)-NPLs modulates its uptake. Considering that the content of Ti in PET(Ti)-NPLs is approximately 2.6% [9,10], a similar amount (3 µg/mL) of TiO_2_-NPs was used to determine its internalization, in comparison with a high concentration (100 µg/mL). As indicated in Figure 3e, the low concentration of TiO_2_-NPs showed a very poor uptake, and a high concentration was required to obtain a percentage of cells with internalized TiO_2_-NPs, like that observed for PET(Ti)-NPLs. When the number of internalized TiO_2_-NPs was determined (Figure 3f), similar results were observed. This indicated that the cell uptake results are not related to the TiO_2_-NP content but rather to the composite nature of PET(Ti)-NPLs.

**Figure 4:**
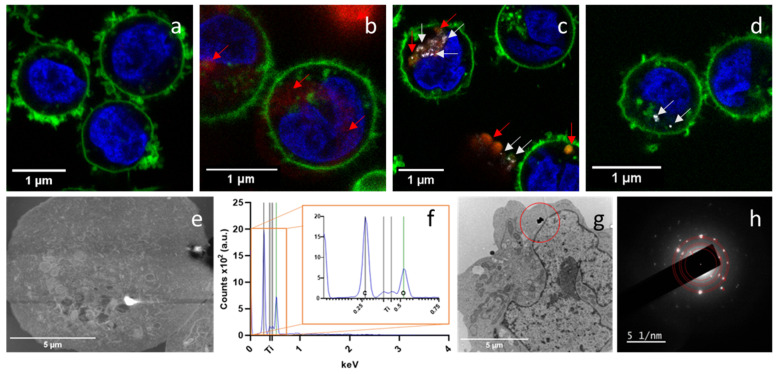
Confocal microscopy images of particle internalization of THP-1 cells using no particles as negative control (**a**), iDye-marked PET-NPLs showing the signal of PET-NPLs in red highlighted with red arrows (**b**), iDye-marked PET(Ti)-NPLs showing the colocalization of signals of Ti as a white signal with white arrows and the PET polymer as a red signal with red arrows (**c**), and TiO_2_NPs as observed on a white signal with white arrows (**d**). Scanning electron microscopy images showing an image of PET(Ti)-NPLs internalized by THP-1 cells (**e**) and the signal translated into an energy dispersive diagram (**f**). Internalized PET(Ti)-NPLs by TEM as indicated by a red circle (**g**) and the diffraction pattern of TiO_2_ contained in the PET(Ti)-NPLs (**h**).

To better visualize the described internalization, complementary methods were also used. Thus, when confocal microscopy was used, the internalization of PET(Ti)-NPLs in THP1 cells exposed to 100 µg/mL for 24 h was observed (Figure 4c). For a better comparison, cells without exposure (Figure 4a), cells exposed to 100 µg/mL of PET-NPLs (Figure 4b), and cells exposed to TiO_2_-NPs (Figure 4d) were also included. Scanning electron microscopy (SEM) methodologies were also used to detect internalization thanks to the electron density of Ti (Figure 4e). The confirmation of this internalization was determined by using energy dispersive X-ray spectroscopy tools identifying the presence of Ti (Figure 4f). Transmission electron microscopy (TEM) was also used to confirm the presence of internalization (Figure 4g), and the use of the electron diffraction pattern (Figure 4h) confirmed the crystalline nature of the Ti-doped PET nanoplastic.

Our results confirm the internalization of PET(Ti)-NPLs and show the advantages of using a wide range of complementary methodologies for detection. Nevertheless, other techniques can also be useful for detecting cell internalization, and among them, Raman microscopy stands out, as demonstrated in the identification of MNPLs in complex biological matrices, including cells [26,27,28]. In addition, Raman scattering signals are consistently stronger when the analyzed particles are not perfectly shaped, as is the case of true-to-life MNPLs [29].

### 3.4. Cell Sorting and Mass Spectroscopy

Dosimetry is a critical factor when evaluating the toxicity of NPLs, especially considering that particulate matter behaves quite differently from chemicals, which always distribute homogeneously in the water column or medium. This makes it essential to describe how the internal dose leads to significant biological changes, since in scavenger cells macrophage alterations in immune-related functions are directly associated with the dose and size of internalized MNPLs [25]. Therefore, estimating the amount of nanoplastics within cells can provide a comparative advantage in drawing final conclusions about the induced effects. In such a context, the use of metal-doped NPLs, as in our case, can permit them to go further than mere visualization. To quantify the number of particles within the cells, we employed cell sorting to use only cells that had internalized PET(Ti)-NPLs.

**Figure 5:**
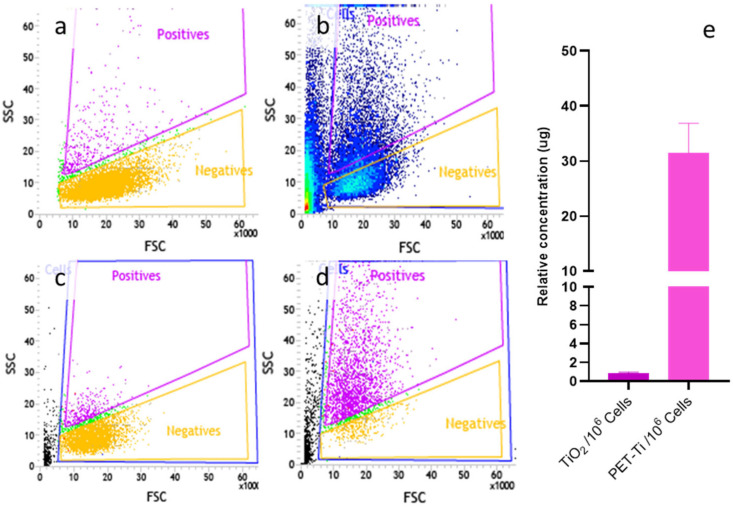
Flow cytometry data on THP-1 of untreated cells (**a**) and cells treated with PET(Ti)NPLs (**b**). After sorting, the number of complex cells is reduced in the negative population of THP-1 cells (**c**), but it is large in THP-1 cells that have internalized PET(Ti)NPLs (**d**). The relative amount of TiO_2_NPs and PET(Ti)NPLs, per million cells, is depicted (**e**).

Using side light scattering, we successfully separated populations according to their complexity. As shown in Figure 5a, some untreated cells exhibited greater complexity than the rest, which must be considered in further analysis. As expected, when cells were treated with PET(Ti)-NPLs, the number of cells shifting to a more complex region increased considerably because of the exposure (Figure 5b). After performing cell sorting, there was a considerably lower number of complex cells in the control population than in the population that had indeed internalized the nanoplastics (Figure 5c,d, respectively). It was observed that even after cell sorting, some cells were still categorized as negative. This could be due to cells that had released internalized PET(Ti)-NPLs into the surrounding medium.

Despite that noise, cell sorting can be a suitable tool to determine the effects associated with MNPL exposure using only those cells that have internalized MNPLs and avoiding the use of cell populations with an undefined percentage of cells with no internalized particles, with a considerable possibility of masking measurable real effects on the truly exposed population. These complex scenarios were observed in whole human blood of healthy donors, where no effects were detectable after MNPL exposure in the complex mixture of blood cell types. Nevertheless, after sorting the different types of white blood cells, significant differences in both internalization and effects were observed between them [19,30]. This type of approach has also been used in mouse alveolar macrophages (MH-S) exposed to PET-NPLs obtained from the degradation of plastic water bottles. That study focused only on the cells that had internalized them as a novel approach capturing the realistic adverse effects only in the cells that have internalized the agent under study, thereby mitigating any biases while assessing the risk of these MNPLs [21].

Interestingly, by determining the amount of titanium in 1 mL of medium containing one million cells, we estimated the presence of approximately 0.82 µg of Ti. Considering that titanium constitutes 2.6% of the used nanoplastics, this value can be estimated at 31.56 µg of PET(Ti)-NPLs per 1 × 10^6^ of THP1 cells, as depicted in Figure 5e. This type of dosimetry only can be carried out using metal-doped NPLs and, although some synthetic metal-doped NPLs have been obtained using different approaches [31,32], our true-to-life PET(Ti)-NPLs can be considered a real representative of the environmental secondary NPLs resulting from the constant degradation of plastic goods.

### 3.5. Reactive Oxygen Species Related to PET(Ti)-NPL and TiO_2_-NP Exposure

**Figure 6:**
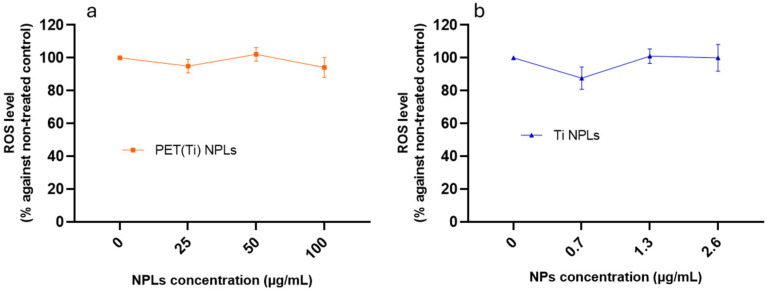
Change in ROS levels after 24 h of THP-1 exposure to 0–100 µg/mL of (**a**) PET(Ti)-NPLs and (**b**) the corresponding concentration of TiO_2_-NPs in the NPLs (0–2.6 µg/mL). Values of ROS were normalized against those in non-treated THP-1 after 24 h.

The induction of intracellular reactive oxygen species (ROS) is considered a common hazard associated with nanoparticle exposure, and increased ROS levels have been reported in different in vivo and in vitro studies after exposure to MNPLs [33]. In our study, no significant changes in the ROS pattern expression were observed (Figure 6). However, it is important to keep in mind that not only is the cell type used for studies relevant but also the systemic response evaluation, as observed in previous studies conducted on *Drosophila* hemocytes, where relevant increases in oxidative damage induction were observed for PET-NPL, PET(Ti)-NPL, and TiO_2_-NP exposure [10,18]. Therefore, a more careful evaluation is needed to unmask possible biological detrimental interactions of the tested NPLs.

### 3.6. DNA Damage Induction

**Figure 7:**
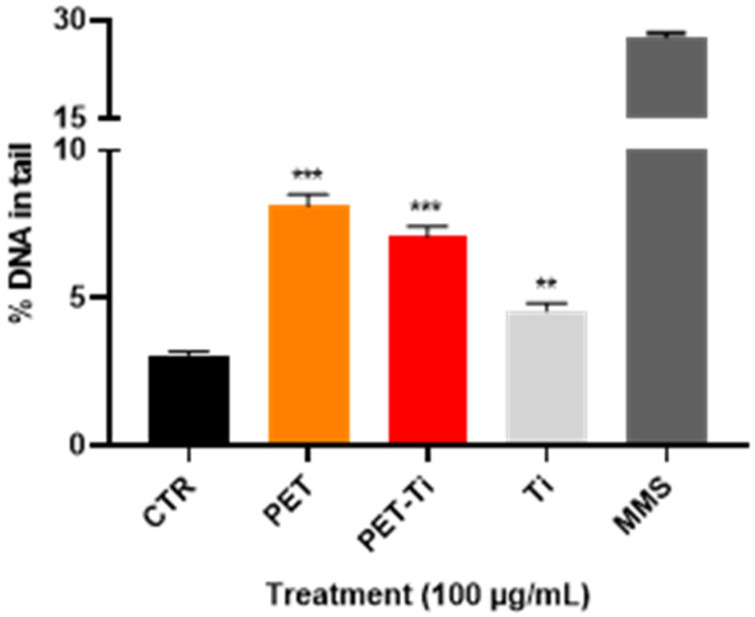
Percentage of DNA on the tail for the comet assay with methyl methanesulfonate (MMS) as a positive control. The effects were induced on THP-1 cells treated for 24 h with 100 µg/mL of PET-NPLs, PET(Ti)-NPLs, and TiO_2_ -NPs. CTR refers to untreated cells as a negative control. ** *p* ≤ 0.01; *** *p* ≤ 0.001.

Aiming to detect the potential harmful effects of PET(Ti)-NPLs, this work focused on their potential genotoxic effects. This is because some alarms exist about the genotoxicity of TiO_2_-NPs. Although titanium compounds, including TiO_2_-NPs, were authorized as a food additive (E 171) in the EU a long time ago, the recent position of the European Food Safety Authority (EFSA) announcing that it is not possible to rule out the genotoxicity from titanium dioxide and therefore that it “can no longer be considered as safe when used as a food additive” [34] suggests that the EU has banned its use [35]. In addition, it is well known that there exists a strong association between DNA damage induction and drastic health consequences for humans [36]. Since DNA damage can result from multiple mechanisms, we selected the comet assay as a tool for detecting primary DNA damage. In our study, PET- and PET(Ti)-NPLs not only internalized but also exhibited genotoxic effects, as shown in Figure 7.

To determine the relative relevance of the two moieties (TiO_2_-NPs and PET-NPLs), THP-1 cells were also exposed to these two components. It is important to note that genotoxic DNA damage was observable in all three cases: PET-NPLs, PET(Ti)-NPLs, and TiO_2_-NPs alone. PET-NPLs alone caused the most significant damage, while PET(Ti)-NPLs were right in the middle of the effects exerted by the PET-NPLs and TiO_2_-NPs alone. High concentrations of PET nanoplastics have been shown in recent studies to induce cell-transforming biological effects, with the potential to promote tumors. However, this effect was only observed at concentrations above 200 µg/mL [20]. On the other hand, genotoxic damage at the dose investigated here has been reported for PET-NPLs in *Drosophila* [17]. Moreover, both PET(Ti)-NPLs and TiO_2_-NPs alone have been shown to effectively alter genotoxic damage pathways in the same model [10]. This, far from being contradictory, may be an indicator of a more systemic reaction than cellular alone. It is important to consider that although both PET-NPLs and TiO_2_-NPs alone showed detectable effects in the comet assay, these effects were not exacerbated by their presence together in a nanoplastic composite. Instead, the effect was diminished. While it is challenging to directly attribute this outcome to a specific cause, it is not difficult to understand that the effect generated by PET-NPLs may have been reduced due to the PET mass reduction due to the additional weight contributed by the Ti in the particle. Thus, our results show that genotoxic damage was readily observed in cell populations exposed to PET-NPLs. It is also evident that PET(Ti)-NPLs induced a similar but less pronounced effect, whereas the damage caused by TiO_2_ NPs alone was detectable by the comet assay. Overall, our findings highlight that PET-NPLs, specifically true-to-life particles, are significant inducers of primary DNA damage in the human immune cell model presented in this study.

## 4. Conclusions

The present study demonstrates that both PET and titanium-doped PET nanoplastics are internalized by THP-1 monocytes. Furthermore, we have demonstrated that the dynamic of uptake is significantly influenced by both particle concentration and exposure duration. The titanium additive in PET(Ti)-NPLs facilitates not only tracking but also dosimetry studies. The presence of titanium appears to mitigate the extent of genotoxic damage compared to PET-NPLs alone. These results underscore the importance of using representative NPL models in toxicological assessments and provide information on the way to future and more complex studies aimed at elucidating the molecular mechanisms underlaying nanoplastic-induced cellular effects.

## Data Availability

Data are contained within the article and Supplementary Materials.

## Supplementary Materials

The following supporting information can be downloaded at: https://www.mdpi.com/article/10.3390/nano15131040/s1, Figure S1: Transmission electron microscopy (TEM) micrographs of PET and PET(Ti) particles are shown (a,d). The hydrodynamic behavior of PET nanoplastics (NPLs) exhibits an average size of 282 nm, a relatively high polydispersity index of 0.47, and a ζ-Potential close to 29 mV (b). Additionally, the correlation coefficient of independent measurements is presented (c). Similarly, PET(Ti) displays an average particle size of approximately 320 nm with a polydispersity index of 0.34 and a ζ-Potential around 18.2 mV. A corresponding correlation curve is provided alongside the graphs (f). Finally, the Fourier-transform infrared spectroscopy (FTIR) spectra of PET and PET(Ti) reveal the characteristic peaks of PET. Interactions of polar ester groups and benzene rings (1), Vibrations of adjacent two aromatic H in p-substituted compound and aromatics bands (2), aromatic rings (3), methylene group aand vibrations of the ester C-O bond (4), Therephtalate group (OOCC_6_H_4_-COO)(5), stretching of the C-O group deformation of the O-H group and bending and wagging vibrational modes of the ethylene glycol segment (6), vibration aromatic skeleton with stretching C=C (7), stretching of C=O of carboxylic acid group (8), axial symmetrical deformation of CO_2_ (9), C-H symmetrical stretching (10), symmetrical stretch of CH (11) and OH (hydroxyl) group (12); Table S1: Material for TEM visualization. Dehydration steps of increasing concentrations of acetone; Table S2: Material for TEM visualization. Steps for resin infiltration at room temperature.
