# Supplementary material for: Biological Impact of True-to-Life PET and Titanium-Doped PET Nanoplastics on Human-Derived Monocyte (THP-1) Cells
Source: Nanomaterials (Basel). 2025 Jul 4;15(13):1040. doi: 10.3390/nano15131040 (PMC12250938; doi:10.3390/nano15131040)
Supplement: Supplementary file 1 [file nanomaterials-15-01040-s001.zip › nanomaterials-3727073-supplementary.pdf]

Biological Impact of True-To-Life PET and Titanium-Doped PET Nanoplastics on Human-Derived Monocyte (THP-1) Cells

Aliro Villacorta [1,2,*,†], Michelle Morataya-Reyes [1,†], Lourdes Vela [1,3], Jéssica Arribas Arranz [1], Joan Martín-Perez [1], Irene Barguilla [1], Ricard Marcos [1] and Alba Hernández [1,*]

[1] Group of Mutagenesis, Department of Genetics and Microbiology, Faculty of Biosciences, Universitat Autònoma de Barcelona, 08193 Cerdanyola del Vallès, Spain; herlem.morataya@uab.cat (M.M.-R.); lulis002@hotmail.com (L.V.); jessica.arribas@uab.cat (J.A.A.); juan.martin.perez@uab.cat (J.M.-P.); irene.barguilla@uab.cat (I.B.); ricard.marcos@uab.cat (R.M.)

[2] Facultad de Recursos Naturales Renovables, Universidad Arturo Prat, Iquique 101000, Chile

[3] Faculty of Health Sciences Eugenio Espejo, Universidad UTE, Quito 170508, Ecuador

[*] Correspondence: avillaco@unap.cl (A.V.); alba.hernandez@uab.cat (A.H.)

[†] These authors contributed equally to this work.

SUPLEMENTARY MATERIAL

S1. Complementary characterization studies

S1.1. Dynamic light scattering (DLS) & ζ-Potential

From the nanogenotox dispersed nanoplastic, 50 µg/mL dilution were prepared. Each suspension was then homogenized by vortex and then 1.5 millilitre was transferred to corresponding DTS0012 cuvettes for size determination and a DTS1070 cuvette was filled for ζ-Potential measurements. In all cases the refractive index used was 1.58 and the light scattering collection angle was 174.8. All measurements were conducted on a Zetasizer Nano ZS and data was collected with a Zetasizer software Version 7.12 both from Malvern Panalytical Ltd (Cambridge, United Kingdom).

S1.2. Transmission electron microscopy (TEM)

Transmission electron micrographs were obtained on a JEOL JEM 1400 microscope (Instrument JEOL LTD, Tokyo, Japan). For sample preparation, working solutions at concentrations of 100 µg/mL were prepared from stock. A single 12 µL drop of PET & PET(Ti) NPLs was transferred to individual carbon covered cooper grids and let dry overnight on filter paper covered petri dishes inside a laminar air flow hood. Particles were then analyzed on a TEM Hitachi H-7000 (Hitachi Ltd., Tokyo, Japan). Micrographs were taken from random fields.

S1.3. Fourier transform infrared spectroscopy (FTIR)

Samples were prepared by resuspending the corresponding particles powder in MiliQ water by vortex to a final concentration of 10,000 µg/mL and then pipetting a 10 µL drop into a gold mirror holder. Samples were dried for a week inside a closed petri dish to prevent contamination and then the analysis of organic functional groups emission of nanoplastics was analyzed using a Hyperion 2000 from Bruker corporation (Bruker Corp., Billerica, MA, USA).

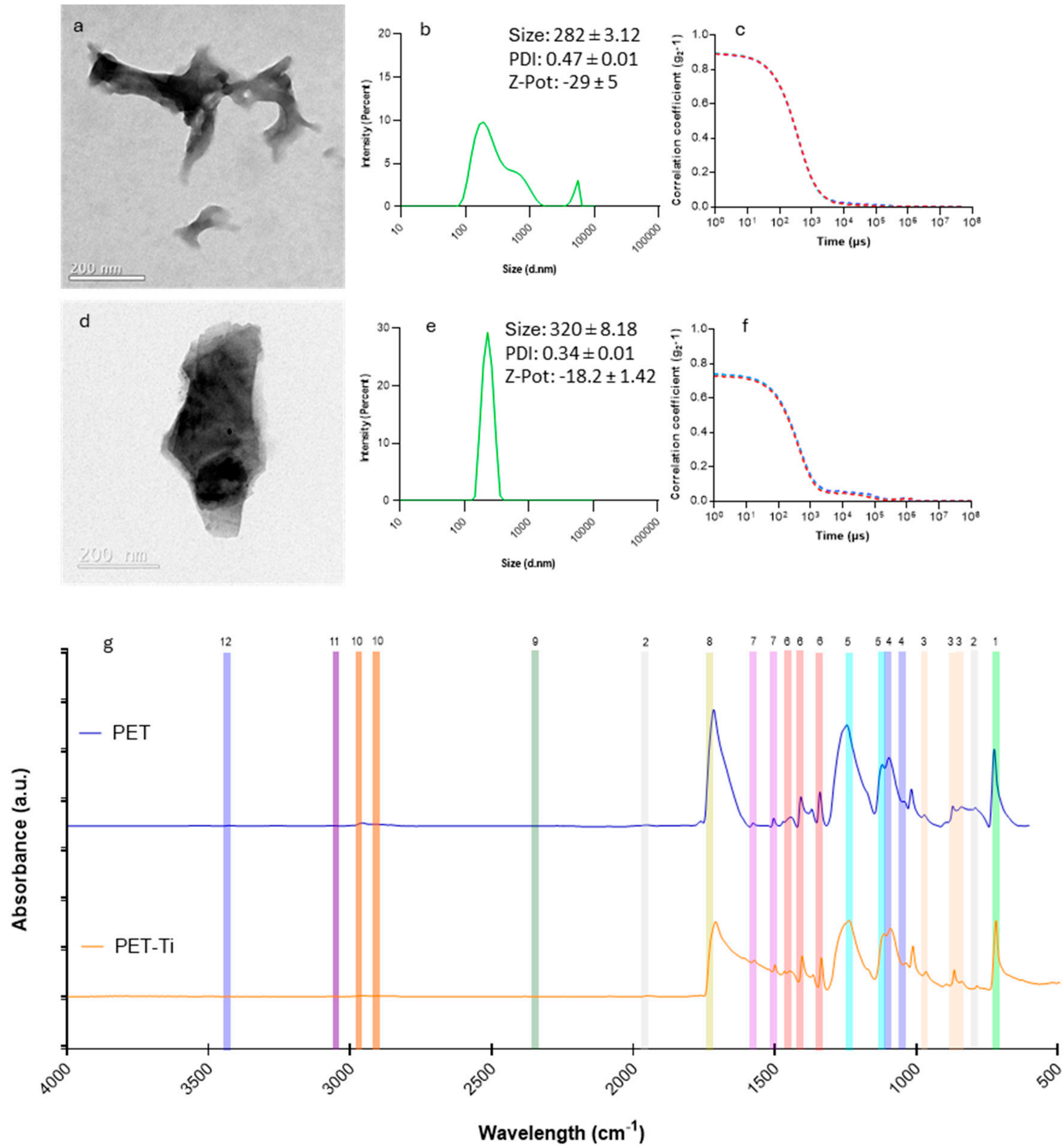

Figure S1. Transmission electron microscopy (TEM) micrographs of PET and PET(Ti) particles are shown (a & d). The hydrodynamic behavior of PET nanoplastics (NPLs) exhibits an average size of 282 nm, a relatively high polydispersity index of 0.47, and a ζ-Potential close to 29 mV (b). Additionally, the correlation coefficient of independent measurements is presented (c). Similarly, PET(Ti) displays an average particle size of approximately 320 nm with a polydispersity index of 0.34 and a ζ-Potential around 18.2 mV. A corresponding correlation curve is provided alongside the graphs (f). Finally, the Fourier-transform infrared spectroscopy (FTIR) spectra of PET and PET(Ti) reveal the characteristic peaks of PET. Interactions of polar ester groups and benzene rings (1), Vibrations of adjacent two aromatic H in p-substituted compound and aromatics bands (2), aromatic rings (3), methylene group aand vibrations of the ester C-O bond (4), Therephtalate group ($OOCC_6H_4$-COO)(5), stretching of the C-O group deformation of the

O-H group and bending and wagging vibrational modes of the ethylene glycol segment (6), vibration aromatic skeleton with stretching C=C (7), stretching of C=O of carboxylic acid group (8), axial symmetrical deformation of CO_2 (9), C-H symmetrical stretching (10), symmetrical stretch of CH (11) and OH (hydroxyl) group (12).

Table S1. Material for TEM visualization. Dehydration steps of increasing concentrations of acetone

% of acetone	Times	Time (minutes)
50	1x	8
70	2x	10
90	3x	10
96	3x	10
100	2x	12
100	1x	20

Table S2. Material for TEM visualization. Steps for resin infiltration at room temperature

EPON/ACETONE ratio	Times	Time (hours)
1/3	1x	1.2
1/1	1x	1.5
3/1	1x	Over night
Pure EPON	2x	2
Pure EPON	1x	Over night
